# Impact of the Implementation of New WHO Diagnostic Criteria for Gestational Diabetes Mellitus on Prevalence and Perinatal Outcomes: A Population-Based Study

**DOI:** 10.1155/2016/2670912

**Published:** 2016-12-21

**Authors:** Katja Erjavec, Tamara Poljičanin, Ratko Matijević

**Affiliations:** ^1^Department of Obstetrics and Gynecology, University Hospital Merkur, Zajčeva 19, 10 000 Zagreb, Croatia; ^2^Department of Medical Informatics and Biostatistics, Croatian Institute of Public Health, Rockefellerova 7, 10 000 Zagreb, Croatia; ^3^Department of Obstetrics and Gynecology, School of Medicine, University of Zagreb, Šalata 3, 10 000 Zagreb, Croatia

## Abstract

*Objectives*. To determine the impact of the implementation of new WHO diagnostic criteria for gestational diabetes mellitus (GDM) on prevalence, predictors, and perinatal outcomes in Croatian population.* Methods*. A cross-sectional study was performed using data from medical birth certificates collected in 2010 and 2014. Data collected include age, height, and weight before and at the end of pregnancy, while perinatal outcome was assessed by onset of labor, mode of delivery, and Apgar score.* Results*. A total of 81.748 deliveries and 83.198 newborns were analysed. Prevalence of GDM increased from 2.2% in 2010 to 4.7% in 2014. GDM was a significant predictor of low Apgar score (OR 1.656), labor induction (OR 2.068), and caesarean section (OR 1.567) in 2010, while in 2014 GD was predictive for labor induction (OR 1.715) and caesarean section (OR 1.458) only. Age was predictive for labor induction only in 2014 and for caesarean section in both years, while BMI before pregnancy was predictive for all observed perinatal outcomes in both years.* Conclusions*. Despite implementation of new guidelines, GDM remains burdened with increased risk of labor induction and caesarean section, but no longer with low Apgar score, while BMI remains an important predictor for all three perinatal outcomes.

## 1. Introduction

Gestational diabetes mellitus (GDM) significantly contributes to perinatal mortality and morbidity. It has increasing prevalence worldwide [1] and imposes a significant economic burden with important short-term and long-term consequences for the mother and her baby [2]. Women with GDM have 3 to 4 times higher risk of metabolic syndrome later in life [3] and a two times higher risk of developing type 2 diabetes [4]. Children born from pregnancies complicated with GDM also seem to have an increased risk of obesity, altered carbohydrate metabolism, and abdominal adiposity during childhood and adolescence [5–7], although evidence might still be inconsistent [8].

GDM is defined as carbohydrate intolerance of variable severity with onset or first recognition during pregnancy that does not meet the diagnostic criteria of overt diabetes [9]. Present national guidelines in Croatia for diagnosis and management of GDM are based on the recommendation of the International Association of the Diabetes in Pregnancy Study Group (IADPSG) and are in use since 2011 [10]. The same criteria for GDM diagnosis have been used worldwide ever since publication of the HAPO study in 2008 [11], culminating with publication of new WHO guidelines for diagnosis of GDM in 2013 [12]. Before that period, Croatian national guidelines for GDM diagnosis and management were using the 1999 WHO criteria [13]. Those two guidelines have not been compared regarding their efficacy, but a recently published report estimated that new criteria will increase two- to threefold the number of women diagnosed with GDM during pregnancy, with unclear benefits [14].

In order to assess the current situation regarding GDM in Croatia and the potential impact of new diagnostic GDM criteria on perinatal outcome, a retrospective study was conducted and women diagnosed with GDM in 2010 with the 1999 WHO diagnostic criteria were compared to GDM women in 2015 diagnosed using the new WHO criteria of 2013.

## 2. Materials and Methods

This cross-sectional study was performed using data from medical birth certificates (MBC) collected in 2010 and 2014 by Croatian Institute of Public Health (CIPH) as a part of mandatory national perinatal statistics data reporting.

Named years were selected because we believe that they present two different populations of pregnant women concerning diagnosis and management of GDM. GDM care throughout the country is defined by national guidelines published by the perinatal society and it is presumed to be the same regarding diagnosis and management in all centers. GDM screening is suggested for all pregnant women in second trimester by glucose tolerance test in a one-step manner.

The first group delivered in 2010 was selected as representative of pregnant women diagnosed and managed for GDM using the 1999 WHO criteria where cut-off values after intake of the 75 g OGTT were fasting glucose value ≤ 6.1 mmol/L and 2-hour glucose value ≤ 7.8 mmol/L [13]. These criteria were used in Croatia until 2011 when they were changed with current national guidelines. For comparison group we opted for year 2014 when all perinatal units changed their guidelines to those defined by the HAPO study and the International Association of Diabetes and Pregnancy Study Group (IADPSG) [10], recommending one-step 75 g OGTT test at 24–28 weeks for women not previously diagnosed with overt diabetes. GDM is diagnosed if plasma glucose values meet or exceed fasting value ≤ 5.1 mmol/L, 1-hour value ≤10.0 mmol/L, and 2-hour value of ≤8.5 mmol/L [15, 16].

Data from MBC used in this study consists of maternal data (age, height, and weight before and at the end of pregnancy), antenatal and perinatal issues (presence of GDM, onset of delivery, and mode of delivery), and neonatal data (birth weight and five-minute Apgar score). In order to compare two periods and consequently two diagnostic GDM policies we also assessed selected data concerning GDM incidence.

Primary objective of this study was to determine the incidence of GDM in Croatian population before and after implementation of new guidelines. Secondary objectives assessed the influence of GDM on labor outcome (birth weight and proportion of newborns in three weight categories: <2500 g, between 2500 and 4000 g, and >4000 g, incidence of 5-minute Apgar score <7, induction of labor, and caesarean section rate) and maternal risk factors for GDM (age, prepregnancy BMI, and weight gain during pregnancy) again, before and after implementation of new guidelines.

All statistical analyses were performed using STATISTICA ver. 12.0. Normality of distribution was tested using Shapiro-Wilks test, while homogeneity of variance was tested using Levene test. Differences between groups of independent continuous variables were analysed using Kruskal-Wallis test and test of multiple comparison for post hoc comparison, while differences in the occurrence of individual conditions were compared using the chi^2^-test. Logistic regression (LR) analysis was performed for prediction of the probability of low Apgar score, induction of labor, and caesarean section rate. The predictors included in the regression analyses were age, body mass index (BMI) before pregnancy, and diagnosis of GD. An error threshold of *α* = 0.05 was used in the interpretation of the results.

Ethical approval for the study was obtained from CIPH Ethical Committee for Public Health Researches grant number 80-437/1-16. Informed consent was not needed for the study.

## 3. Results

A total of 81.826 deliveries with 84.537 newborns in years 2010 and 2014 together had been reported through MBC to CIPH. If one or more piece of data analysed in this study were missing for some individual, they were excluded from further assessment leaving 81.748 deliveries and 83.198 newborns analysed in this study.

The number of deliveries decreased by 8.3% from year 2010 to 2014. The incidence of GDM was more than double from 2.2% in 2010 to 4.7% in 2014.

Associated factors for GDM are presented in Table 1. In general, women with GDM were significantly older, being more overweight before pregnancy, but gained less weight during pregnancy in both years compared to the rest of the pregnant population in Croatia (Table 1).

Differences in age, BMI, and weight gain between women with and without GDM in 2010 and in 2014 were statistically significant (all *p*'s < 0.001, Kruskal-Wallis test). However, results of multiple comparison showed no difference in those parameters between women with GDM in 2010 and 2014 (test of multiple comparison). Also, differences in rates of newborns with birthweight < 2500 g or above 4000 g were statistically significant between women with and without GDM in 2010 and in 2014 (all *p*'s < 0.001), but no difference was shown in these parameters between women with GDM in 2010 and 2014 (*p* = 0.230, chi^2^-test).

In order to compare the influence of GDM and diagnostic criteria used on labor outcome multivariate logistic regression (MVLR) models with low Apgar score, induction of labor and caesarean section rate were built. MVLR model revealed that the risk for low Apgar score after 5 minutes did not differ significantly between 2010 and 2014. When years were analysed separately, MVLR models suggested that GDM was a significant predictor of low Apgar score in 2010 (*p* = 0.047), but not in 2014 (*p* = 0.330), meaning that the risk of low Apgar score was significantly higher among newborns of women with GDM compared to newborns of women without GDM in 2010 but not in 2014. BMI before pregnancy was a significant predictor of low Apgar score in both years (*p* < 0.001 in 2010 and *p* = 0.001 in 2014) while maternal age was not (*p* = 0.419 in 2010 and *p* = 0.337 in 2014). Children of women with higher BMI had a significantly higher chance to have low Apgar score compared to children of women with lower BMI in both years. By rise of BMI of 1 kg/m^2^ the chance of having low Apgar score increased for 1.5–6%. The chance of low Apgar score did not differ among women in different age groups in neither year. There was no difference concerning number of newborns with low Apgar score and women's age in general population comparing 2010 and 2014.

MVLR models also suggest that GDM and BMI before pregnancy were significant predictors for induction of labor in both years (all *p*'s < 0.001) while maternal age was a significant predictor only in 2014 but not in 2010 (*p* = 0.057 in 2010 and *p* = 0.008 in 2014), meaning that labor was induced more often among women with GDM or higher BMI compared to the rest of Croatian pregnant population in both years, but for older women only in 2014, not in 2010. Assessed year was not a significant predictor for induction of labor (*p* = 0.111) as the induction of labor incidence was similar in 2010 and 2014.

By MVLR models, all three assessed parameters (age, GDM, and BMI before pregnancy) were found to be predictors of caesarean section delivery in both 2010 and 2014 (all *p*'s < 0.001), meaning that women with GDM, higher BMI, and older age had a significantly higher risk of having a caesarean section compared to the rest of the pregnant population. Assessed year again was not a significant predictor of caesarean section (*p* = 0.396), meaning that despite significantly higher GDM prevalence in 2014 compared to 2010, there was no increase of caesarean section risk in GDM group. Results of multivariate logistic regression analysis for low Apgar score, induction of labor, and caesarean section as outcomes are presented in Table 2.

## 4. Discussion

Perinatal data reporting through MBC organized and collected by CIPH has a long history in Croatia giving us an opportunity to assess and analyse national perinatal statistics. It is mandatory that every single birth in the country is recorded in this registry from all delivery units both in 2010 and in 2014. Therefore, all birth centers included in the data from 2010 are also included in the data from 2014, since it is mandatory for each center in the country to report to the CIPH through MBC.

To the best of our knowledge, the diagnosis and management of GDM should be the same in all centers around the country and are defined by national recommendations [15, 16]. Possible avoidance of universal screening, underreporting, and minor variabilities in local policies of GDM management (i.e., induction of labor, etc.) represent the weaknesses of our study. However, all these factors were present in both analysed years and were not significantly altered during the period between 2010 and 2014 and we would not consider them to significantly interfere with the validity of our results.

Prevalence of GDM in Croatia has risen more than two times from year 2010 to 2014. However, it is still lower compared to other developed countries but comparable to some retrospective studies such as the one of Meek at al. [17] Furthermore, a tertiary referral center in Croatia GDM prevalence is reported to be above 20% [18].

Rising prevalence of GDM is a well-known trend observed in the majority of countries worldwide. There are several possible reasons for that. Rise in incidence of obesity [19] as well as older maternal age [20] observed in recent years, being main risk factors associated with GDM [21], gives one possible explanation for increasing numbers of pregnancies being burdened with GDM. Our results confirm this observation as we demonstrated that women with GDM are older and have higher BMIs but gain less weight during pregnancy no matter which criterion is used for diagnosis and management of GDM. However, new GDM guidelines and lower glucose cut-off values surely also influenced GDM prevalence as by new criteria, a substantial number of women were classified to have GDM that would be considered normal according to old criteria.

It was noted that GDM significantly influenced the incidence of low Apgar score after 5 minutes in 2010 but not in 2014. This finding can be interpreted in two ways. One explanation is that, by lowering the threshold for diagnosis of GDM with new criteria, more women were classified to have GDM that were otherwise considered without GDM, so “less severe cases” with better prognosis in 2014 affected the results and presented better overall outcome. On the other hand, rise of awareness of GDM in the past few years leads to better diagnosis, management and appropriate timing, and mode of delivery of women with GDM that may consequently influenced the incidence of low Apgar score in 2014. Still, there was no difference in perinatal mortality between years, being 4.7/1000 in 2010 [22] and 4.2/1000 in 2014 [23].

To support that, GDM was found to influence both induction of labor and caesarean section rate in both years. This was not influenced by diagnostic criteria used and was independent of age and prepregnancy BMI of women included in this study. Women with GDM had a 50% higher risk of having a caesarean section and a more than double risk of labor being induced compared to women without GDM in both years. However, the chance for both outcomes previously listed was higher in 2010 than in 2014. GDM is traditionally associated with increased rate of caesarean deliveries [24], but only according to data reported before results of HAPO study in 2010 and before new WHO guidelines for GDM in 2013, while studies on perinatal outcomes after implementation of new GDM guidelines are scarce. This suggests that despite significant increase in prevalence of GDM, the prevalence of GDM related induction of labor and caesarean section has decreased, highlighting improvements in management of GDM in recent years. Also, comparing years 2010 and 2014 it is clear that overall incidence of caesarean section and induction of labor rose in Croatian pregnant population [22, 23]. Therefore, introduction of new guidelines defined by the HAPO study and the International Association of Diabetes and Pregnancy Study Group (IADPSG) [10] had positive influence on induction of labor and caesarean section rate in Croatian GDM population. Children of women with GDM had less often normal birth weight (2500–4000 g) and more often low (<2500 g) or high birth weight (>4000 g) compared to children of women without GDM in both years. The rate of macrosomia (newborns born with more than 4000 g) among women with GDM was similar in both years (17.23% in 2010 and 16.75% in 2014); hence we do not consider macrosomia to have significantly influenced the results presented in this study.

Absence of difference in age, BMI, and weight gain between women with GDM in 2010 and 2014 (test of multiple comparison) means that the population of pregnant women with GDM is so strongly associated with these risk factors that even more strict glucose cut-off criteria did not change this association. BMI before pregnancy is an important predictor for all three perinatal outcomes analysed in this study. Higher incidence of low Apgar score might be explained by increased chance of pregnancy complications for overweight and obese women including preeclampsia, gestational hypertension, gestational diabetes, and macrosomia [25, 26], as an indirect cause of adverse neonatal morbidities. Also, neonates born to obese women have an increased risk of birth defects and neonatal hypoglycemia [27]. However, newborns of women with GDM had significantly lower Apgar score in 2010 compared to 2014. This might be influenced, among others, not only by improvement of perinatal care but also by diagnostic criteria used for selecting GDM population. By more intensive surveillance, closer follow-up, and appropriate timing of induction of labor, it is possible that we reduced incidence of low Apgar score as adverse perinatal outcome in GDM population.

Increased induction of labor and caesarean section rate among women with higher BMI have already been reported in certain studies [28] and a recent meta-analysis has estimated the risk of caesarean section to be double for obese women and triple for women with severe obesity with BMI > 35 kg/m^2^ [29]. The myometrium of obese women is considered to be less responsive to oxytocin and obese women more often give birth to macrosomic babies potentially being responsible for caesarean section as mode of delivery [30, 31]. By MVLR models women with higher BMI as well as women with GDM had a significantly higher risk of induction of labor and delivery by caesarean section. However, none of these two outcomes were influenced by GDM criteria used in different years. The only difference found comparing two analysed periods was higher proportion of older women with GDM having induction of labor in 2014 compared to 2010, but we were unable to relate this observation to the GDM diagnostic criteria used.

## 5. Conclusions

GDM remains burdened with increased risk of induction of labor and caesarean section rate as well as the incidence of low Apgar score despite implementation of new diagnostic criteria and management guidelines. However, we found GDM to be associated with lower incidence of low 5 min Apgar score in 2014 compared to 2010. This may be influenced by several parameters, but more precise and more strict diagnostic guidelines as well as management adjusted to these guidelines may be indirectly responsible for this observation. Ideally, well-designed randomised controlled trials comparing present and new diagnostic GDM criteria will give us an answer to entirely understand the significance and impact of new diagnostic guidelines on pregnancy outcome.

## Figures and Tables

**Table 1:** Maternal and newborn characteristics of women with and without GDM in Croatia in 2010 and 2014.

	Without GDM 2010	With GDM 2010	Without GDM 2014	With GDM 2014
Pregnant women	*n* = 41703	*n* = 953	*n* = 37263	*n* = 1829
Age (years)^*∗*^	28.77 ± 5.23 (28.72–28.82)	30.88 ± 5.23 (30.55–31.20)	29.49 ± 5.33 (29.44–29.55)	31.34 ± 5.19 (31.10–31.57)
BMI (kg/m^2^)^*∗*^	23.38 ± 3.99 (23.34–23.41)	25.84 ± 528 (25.51–26.18)	23.38 ± 4.11 (23.33–23.42)	26.03 ± 5.64 (25.77–26.29)
Weight gain (kg)^*∗*^	14.51 ± 5.29 (14.46–14.56)	12.57 ± 5.62 (12.21–12.92)	14.19 ± 5.71 (14.14–14.25)	12.50 ± 5.76 (12.24–12.77)
Excessive weight gain (BMI)°				
Underweight (<18.5)	535 (24.6)	6 (26.1)	434 (20.3)	7 (15.9)
Normal weight (18.5–24.99)	9201 (32.8)	112 (23.8)	7401 (30.5)	225 (25.6)
Overweight (25.0–29.9)	5135 (66.1)	147 (55.9)	4468 (63.5)	228 (49.5)
Obese (≥30)	1851 (66.1)	92 (48.7)	1719 (64.7)	185 (47.4)
Obesity class (BMI)^°•^				
I (≥30 kg/m^2^)	2101 (5.0)	127 (13.3)	2021 (5.4)	248 (13.6)
II (≥35 kg/m^2^)	541 (1.3)	49 (5.1)	483 (1.3)	105 (5.7)
III (≥40 kg/m^2^)	156 (0.4)	13 (1.4)	151 (0.4)	37 (2.0)
Newborns	*n* = 42438	*n* = 981	*n* = 37904	*n* = 1875
Birth weight (g)^*∗*^	3401 ± 555 (3396–3407)	3439 ± 621 (3400–3478)	3386 ± 564 (3381–3392)	3455 ± 619 (3427–3483)
Birth weight categories°				
<2500	2019 (4.8)	66 (6.7)	2024 (5.3)	98 (5.2)
2500–4000	35371 (83.4)	746 (76.0)	31625 (83.5)	1463 (78.0)
>4000	5048 (11.9)	169 (17.2)	4245 (11.2)	314 (16.8)

^*∗*^Data are presented as mean ± SD (95 −CI–95 +CI)

°Data are presented as number (%)

^•^Obesity classes are defined according to The International Classification of adult underweight, overweight, and obesity according to BMI. Available at http://www.who.int/mediacentre/factsheets/fs311/en/.

**Table 2:** Multivariate LR models for low Apgar score, induction of labor, and caesarean section as an outcome.

Year	Risk factors	Odds ratio	95% CI	*p*
Low Apgar score				

2010	GDM	1.656	1.008–2.720	0.047
	Age	1.008	0.989–1.028	0.419
	BMI before pregnancy	1.050	1.028–1.073	<0.001

2014	GDM	1.246	0.800–1.939	0.330
	Age	0.991	0.971–1.911	0.337
	BMI before pregnancy	1.038	1.015–1.062	0.001

Induction of labor				

2010	GDM	2.068	1.761–2.427	<0.001
	Age	0.994	0.988–1.000	0.057
	BMI before pregnancy	1.042	1.035–1.050	<0.001

2014	GDM	1.715	1.515–1.940	<0.001
	Age	1.008	1.002–1.014	0.008
	BMI before pregnancy	1.039	1.031–1.046	<0.001

Caesarean section				

2010	GDM	1.567	1.360–1.806	<0.001
	Age	1.040	1.035–1.045	<0.001
	BMI before pregnancy	1.049	1.043–1.055	<0.001

2014	GDM	1.458	1.310–1.622	<0.001
	Age	1.040	1.035–1.045	<0.001
	BMI before pregnancy	1.045	1.039–1.051	<0.001
